# Design and Internal Validation of an AI‐Assisted Clinical Decision Support System (CDSS) for Telemedicine Services to Augment Patient Care in Rural Areas of Rajasthan

**DOI:** 10.1155/ijta/8245320

**Published:** 2026-10-28

**Authors:** Rahul Singh Chowhan, Sumit Kalra, Ramesh Kumar Huda, Pankaj Bhardhwaj

**Affiliations:** ^1^ ICMR-National Institute of Health Research Jodhpur Rajasthan India; ^2^ Department of Computer Science and Engineering Indian Institute of Technology (IIT) Jodhpur Jodhpur Rajasthan India iitp.ac.in

**Keywords:** artificial intelligence, clinical decision support system, doctor-in-the-loop, incremental learning, rural healthcare, telemedicine

## Abstract

**Background:**

Conventional teleconsultation platforms function primarily as communication interfaces and lack integrated clinical intelligence or adaptive learning capability. This study presents the development and design evaluation of the TeleMed Clinical Decision Support System (CDSS v2.5), an AI‐assisted predictive framework engineered to augment teleconsultation quality in rural areas of Rajasthan, India.

**Methods:**

A development and internal validation design following TRIPOD‐AI guidelines was used to develop TeleMed CDSS v2.5. The framework integrated TabPFN 2.5 for tabular prediction, MiniLM embeddings for semantic symptom normalization, and KNN‐based clinical retrieval within an offline‐first, doctor‐in‐the‐loop telemedicine platform. Internal validation used stratified fivefold cross‐validation on the complete 4401‐record dataset. A clinician‐curated 100‐case edge‐case robustness suite, selected from the main dataset and reserved for secondary evaluation, was used to assess performance on linguistic edge cases and multidomain presentations. This suite is not a separate validation cohort to provide independent evidence of generalization. For comparative benchmarking, XGBoost, random forest, logistic regression, and a rule‐based triage model were evaluated using fivefold stratified cross‐validation on the same dataset. All AI‐generated outputs were reviewed, modified if necessary, and approved by the consulting clinician; only parent‐category classification was quantitatively evaluated.

**Results:**

The TeleMed CDSS v2.5 achieved a macro AUROC of 0.962 (95% CI: 0.953–0.971) and accuracy of 87.2% (95% CI: 0.858–0.886) under fivefold stratified cross‐validation. On the clinician‐curated 100‐case edge‐case robustness suite, category‐level accuracy was 90.0% as an internal robustness assessment. Comparator model accuracies under fivefold cross‐validation were: TabPFN 2.5 standalone (0.831), XGBoost (0.845), random forest (0.827), logistic regression (0.792), and rule‐based triage (0.743). The complete multilayer pipeline improved upon standalone TabPFN by 10.7 percentage points on the 100‐case suite (90.0% vs. 79.3%). Contextual adaptation remained stable from the initial 2000‐record model through sequential expansion to 3000, 4000, and 4401 records, with accuracy differences from full retraining below 1 percentage point. The 8000‐record condition was a projected stress‐test and was not empirically observed during the study period.

**Conclusions:**

The TeleMed CDSS v2.5 demonstrates technical feasibility of a hybrid AI framework integrating transformer‐based prediction, semantic symptom normalisation, and doctor‐guided contextual adaptation within a rural telemedicine workflow. These findings are limited to the design‐evaluation phase; external validation and prospective clinical effectiveness studies are required before broader deployment.

**Trial Registration:** CTRI: CTRI/2024/10/076070

## 1. Introduction

Access to timely and equitable healthcare remains a major challenge in geographically isolated and rural communities because of poor infrastructure, workforce shortages, and limited specialist availability. Health inequalities in rural Rajasthan are compounded by sociodemographic disparities, with rural communities, women of reproductive age, and the geriatric population disproportionately underserved because of limited mobility, cultural barriers to care‐seeking, and distance from district health facilities. India′s eSanjeevani platform has facilitated over 150 million teleconsultations since 2020, but teleconsultation quality is constrained by the cognitive burden on doctors managing high patient volumes, limited physical examination, and absence of real‐time decision support tools [1, 2]. In rural India, these barriers often lead to delayed healthcare‐seeking behavior, reliance on informal treatment practices, and worsening health outcomes. Telemedicine integrated with digital diagnostics and artificial intelligence (AI) has emerged as a promising approach for enabling remote consultations and supporting clinical decision‐making in hard‐to‐reach areas. AI‐powered systems can further enhance telemedicine through symptom‐based assistance, intelligent triage, and real‐time clinical support, thereby improving accessibility and continuity of care in low literacy and low connectivity environments [3].

Despite these advancements, conventional telemedicine platforms largely function as communication interfaces and often lack integrated clinical intelligence, adaptive learning capability and decision support. Studies have shown that unassisted telemedicine systems frequently struggle in rural ecosystems because of intermittent connectivity, low digital literacy, and the absence of structured clinical workflows, limiting their effectiveness in supporting frontline healthcare providers [4]. Consequently, there is growing recognition that telemedicine must evolve into an assisted model where intelligent clinical systems augment healthcare providers during consultations, thereby improving diagnostic consistency and treatment efficiency [5–7].

The integration of AI‐driven clinical decision support systems (CDSS) into telemedicine offers a transformative pathway to address these limitations. AI‐enabled CDSS can analyze symptom patterns, physiological parameters, historical clinical data to generate evidence‐based recommendations, prioritize cases, and support personalized care delivery [8]. Such systems can also streamline administrative workflows, optimize resource utilization, and improve remote monitoring, particularly in rural settings with limited specialist oversight [9–11]. However, the real‐world deployment of AI‐assisted predictive telemedicine models remains constrained by challenges related to model adaptability, lack of doctor trust, opaque algorithmic reasoning, and poor integration with existing clinical workflows. Static predictive models trained on homogeneous datasets often underperform in dynamic rural environments characterized by diverse patient presentations and resource variability [12, 13].

Furthermore, successful adoption of AI in telemedicine requires doctor‐in‐the‐loop systems that incorporate doctor feedback, transparent decision‐making, and continuous learning mechanisms to ensure safety, usability, and regulatory compliance [11, 14]. The need for adaptive, context‐aware, and incrementally learning AI systems tailored for low‐resource and geographically isolated regions therefore remains critically underexplored. Evidence from low‐resource deployment settings suggests that adaptive AI systems combining clinical safety rules with continuous physician feedback can meaningfully reduce diagnostic inconsistency and cognitive burden in high‐volume teleconsultation environments [15, 16].

Therefore, to address these gaps this study presents the design, development, and clinical workflow integration of AI‐assisted predictive TeleMed CDSS (CDSS v2.5) for supporting clinical decision‐making and augmenting patient care delivery in rural areas of Rajasthan. The primary objective of this study is to develop and conduct a design evaluation of the TeleMed CDSS v2.5 as a technical feasibility study of a hybrid AI‐CDSS within a doctor‐in‐the‐loop rural telemedicine workflow.

## 2. Methods and Materials

### 2.1. Study Design and Setting

The study was conducted at a single rural telemedicine facility in western Rajasthan. The site served as a hub‐and‐spoke teleconsultation center providing primary care services to patients from surrounding rural communities with limited access to healthcare. This study employed a development and internal validation design, consistent with TRIPOD‐AI reporting guidelines for prediction model studies at the design‐evaluation phase [17]. A participatory, iterative approach was adopted during system development, incorporating structured clinical feedback from consulting doctors to refine interface design, alert thresholds, and output formatting. The three‐layer system architecture is summarized in Table S1.

### 2.2. Study Population

All patients presenting for teleconsultation between September 2024 and March 2025 were included, spanning all age groups and both genders. The final dataset of 4401 teleconsultation records exceeds the minimum sample size of 2080 records for multivariable prediction model development by a factor of 2.1, ensuring adequate representation for stable multiclass coefficient estimation [18].

### 2.3. System Architecture and Technical Design

The TeleMed CDSS v2.5 was developed as an offline‐first (following initial model deployment) lightweight clinical decision support platform for telemedicine use in low‐connectivity settings. The system followed a three‐layer architecture comprising a React 19.2 and TypeScript frontend interface for clinical interaction, a FastAPI and Uvicorn backend (Python 3.10 +) for API handling and model serving, and a machine learning engine integrating TabPFN 2.5, MiniLM embeddings, and scikit‐learn–based retrieval and validation modules. The six‐stage prediction pipeline implemented within the ML engine is described in detail in Section 2.5 and algorithmic details for the novel components is provided in Table S7.

The platform processed structured and free‐text clinical inputs including age, gender, symptoms, symptom duration, blood pressure, SpO₂, and heart rate to generate AI‐assisted predictive diagnostic and treatment recommendations during teleconsultations. The AI‐generated outputs included parent‐category classification, provisional diagnosis, medication prescription suggestions including days to continue medication, treatment advice, behavior, and clinical remarks. All outputs were presented in an editable format and were assessed by the consulting clinician, who could validate, modify, or override them before finalizing the consultation. Symptom normalization was performed using all‐MiniLM‐L6‐v2 sentence transformers (384‐dimensional embeddings), enabling mapping symptom descriptions into standardized clinical terminology through cosine similarity matching. To prevent label leakage, disease‐label tokens detected via cosine similarity > 0.70 against a diagnosis index were explicitly excluded from the symptom vocabulary. The predictive framework utilized TabPFN 2.5, a transformer‐based tabular foundation model optimized for heterogeneous clinical datasets. To overcome the native 10‐class limitation of TabPFN, a decimal positioning strategy was implemented in which broad category prediction and fine‐grained classification were performed in sequential stages and combined probabilistically for calibrated multiclass prediction. A K‐nearest neighbor (KNN) retrieval layer grounded predictions in similar historical clinical records using semantic symptom similarity and normalized physiological parameters, while safety validation rules and domain‐specific weighting were applied to reduce diagnostic ambiguity. The system additionally supported doctor‐guided corrections in AI‐predicted diagnoses and allowed individual or CSV/Excel‐based dataset uploads, enabling continuous incremental learning and adaptation to evolving clinical patterns.

### 2.4. Data Collection and Preprocessing

Patient data were entered into the teleconsultation platform by frontline healthcare workers who had received standardised training in clinical data collection at the ICMR‐NIIRNCD (National Institute for Implementation Research on Noncommunicable Diseases) headquarters, Jodhpur, Rajasthan. This manuscript focuses exclusively on the AI module component of the larger ICMRf‐funded telemedicine project. The dataset included demographic details, symptom profiles, physiological parameters, diagnostic categories, treatment advice, medication recommendations, and doctor remarks relevant to rural primary care settings. Input variables included age, gender, symptoms, symptom duration, systolic and diastolic blood pressure, oxygen saturation (SpO₂), and heart rate. Output variables included diagnosis, parent disease category, advice, medicines, behavior, and clinical remarks. Both structured and free‐text symptom entries were incorporated to reflect real‐world teleconsultation workflows. A complete specification of all input features and output targets with their clinical rationale is provided in Table S2.

Ground‐truth parent‐category labels were generated during routine assisted teleconsultations and were not independently adjudicated. Labels were assigned by MBBS‐qualified consulting doctors involved in the teleconsultation workflow. Three clinicians participated in label generation. Each teleconsultation encounter was labelled by the consulting doctor responsible for that consultation; encounters were not independently double‐labelled. The label represented the final clinical impression recorded at the end of the consultation, after the clinician reviewed the patient history, vital signs, symptom description, and AI‐assisted suggestions.

Corrections were made through the doctor‐validation interface. When the consulting doctor modified or overrode an AI‐generated parent‐category suggestion, the saved modification replaced the AI‐generated output and became the final label used for analysis and contextual adaptation. These corrections were logged within the system. No independent adjudication panel reviewed all labels, and no formal disagreement‐resolution procedure was applied for the main dataset because the treating clinician′s final saved entry was accepted as the operational reference standard. For the clinician‐curated 100‐case robustness suite, expected parent‐category labels were confirmed by consensus among the consulting clinicians. The exact number and demographic characteristics of participating clinicians were removed during deidentification. Records with identical symptom text, vital values, and timestamp were flagged during preprocessing and excluded prior to cross‐validation to prevent exact duplicate encounters from appearing in both training and test folds. Flagged records were reviewed to reduce duplicate‐entry artifacts. As the analytical dataset was deidentified, patient‐level identifiers were not retained; therefore, repeated encounters from the same patient could not be explicitly grouped or excluded. The clinician‐curated 100‐case edge‐case robustness suite was constructed as an internal stress test from the same 4401‐record development corpus. Cases were purposively selected by the consulting clinicians to represent common rural primary‐care presentations, specialized presentations, and edge‐case records. An edge case was defined a priori as a record containing one or more of the following: typographical errors in symptom text; colloquial or nonstandard symptom phrasing; disease‐label contamination in the symptom field; single‐symptom presentations; extreme vital‐sign values; or multidomain diagnostic ambiguity. Case selection was performed before clinicians had reviewed model predictions for the selected records. The clinicians selecting cases had access to model outputs during selection; this is acknowledged as a potential source of selection bias. The expected parent‐category labels were the same final clinician‐verified diagnostic labels used in the main dataset. The suite was fixed before ablation testing and was not used for model fitting, hyperparameter selection, or incremental buffer adaptation. Because the primary fivefold cross‐validation used the full 4401‐record dataset, the 100‐case suite acted as a complementary internal robustness evaluation.

The symptom embedding pipeline encodes only patient‐entered symptom text; diagnostic labels and medication outputs are stored in a separate clinical index and retrieved via distance‐weighted KNN search. Label leakage through the embedding layer is therefore architecturally precluded, though a formal permutation‐based leakage audit is acknowledged as a future validation requirement.

The preprocessing pipelines included:

• Before model development, clinician‐entered free‐text diagnostic labels were normalized using SNOMED CT into a fixed 21‐class parent‐category taxonomy, with compound categories such as “cardiovascular and gastrointestinal” assigned when clinician‐verified diagnoses spanned two parent domains [19].
• Linguistic standardization: MiniLM sentence embeddings via a semantic normalization pipeline were designed to map phonetically varied and colloquial English‐language symptom descriptions to a standardised training vocabulary through sentence‐level cosine similarity matching. Crucially, any tokens exhibiting a cosine similarity > 0.70 to known diagnostic labels were explicitly excluded from the symptom vocabulary to prevent label leakage.
• Temporal structuring: Patient encounters were timestamped and sequentially appended to the incremental learning buffer, enabling a sliding context buffer to maintain recency‐weighted adaptation to evolving local disease patterns.

All records were anonymized at the point of capture, stored locally using encryption in compliance with national data protection standards.

### 2.5. Model Development and Incremental Learning Framework

The TeleMed CDSS v2.5 employed a hybrid AI framework integrating TabPFN 2.5, semantic symptom embedding, and similarity‐based clinical retrieval. It is important to distinguish the adaptation mechanism from conventional gradient‐based continual learning paradigms. The TabPFN classifier weights remained frozen throughout deployment, whereas adaptation was achieved through dynamic contextual refitting by updating the active training context buffer with verified teleconsultation records and refitting the classifier heads at inference without backpropagation. This approach inherently mitigates catastrophic forgetting by design [20].

The six‐stage prediction pipeline operated on the 21‐class parent‐category labels defined via the category normalization step described in Section 2.4. The system comprised a six‐stage prediction pipeline: (1) semantic symptom normalization (cosine similarity threshold ≥ 0.45); (2) TabPFN 2.5 decimal positioning two‐stage decomposition for parent‐category classification across 21 diagnostic classes; (3) capped linear domain boosting (boost = min [1.0 + 0.5 × matching tokens, 3.0]); (4) KNN‐based retrieval for high‐cardinality outputs using a combined scoring function of 0.7 × symptom similarity + 0.3 × (1 − numeric distance); (5) multilayer clinical validation incorporating antibiotic safety flagging and low‐confidence alert generation; and (6) a contextual adaptation stability evaluation was conducted separately. The initial model was developed using 2000 teleconsultation records. Formal stability evaluation was performed after sequential expansion to 3000, 4000, and 4401 records, and was compared against a full retrain baseline. A projected 8000‐record sliding‐window scenario was included as a stress‐test of the FIFO eviction policy; this projected condition was not empirically observed during the study period.

The key pipeline parameters were empirically chosen during internal system development and held fixed for the primary evaluation. These included the semantic normalization threshold of 0.45, the diagnostic‐label exclusion threshold of 0.70, the domain‐boost coefficient of 0.5 capped at 3.0, KNN with k = 5 and scoring weights of 0.7 for symptom similarity and 0.3 for numeric distance, the 8000‐record FIFO context window, and the low‐confidence alert threshold of 0.45. The reported fivefold evaluation used these fixed parameter values.

Doctor corrections were incorporated through the following mechanism: only modifications saved via the doctor validation interface were accepted as feedback signals. Each validated correction triggered an immediate on‐demand contextual refit by rebuilding the symptom index and refitting the TabPFN classifier heads without requiring system downtime. The frozen base weights ensured that no individual correction could induce a catastrophic shift in previously learned patterns. Table S7 provides implementation logic for decimal positioning, MiniLM‐based symptom normalization, contextual buffer refitting, KNN‐based retrieval augmentation, and multilayer clinical validation, including thresholding and invalid‐class handling.

### 2.6. Clinical Workflow Integration

The AI‐predictive model was integrated into the assisted telemedicine workflow as a real‐time clinical decision support tool for consulting doctors. During teleconsultation sessions, patient demographic details, symptoms, symptom duration, and physiological parameters including blood pressure, SpO₂, and heart rate were entered through the clinician‐facing interface. The AI engine, based on submitted data, generated diagnostic suggestions, confidence scores, disease categories, treatment advice, medication recommendations, and clinical remarks. These outputs were presented in an editable format, enabling the consulting doctor to validate, modify, or override the AI‐generated recommendations based on clinical assessment and patient context. The workflow follows a three‐step process:

• Triage and data entry: Frontline healthcare workers recorded vitals and entered symptoms via structured forms with autocomplete and uploaded prior prescriptions or reports.
• AI‐augmented consultation: The system generated structured outputs (diagnosis, confidence, treatment advice, safety alerts, and referral flags) during the live video consultation. Feature importance via perturbation‐based explainability was available if needed.
• Doctor validation and prescription: The remote clinician reviewed AI predictions, finalized the diagnosis, and issued an e‐prescription with follow‐up instructions. All medication outputs required explicit doctor approval before issuance; the system could not generate or send prescriptions autonomously.

### 2.7. Analytical Methods and Validation Framework

The initial AI model was developed using 2000 teleconsultation records collected during the initial telemedicine deployment phase, during which the system was in active development and training. Thereafter, every new verified teleconsultation record was used for incremental contextual adaptation through the sliding buffer mechanism, expanding the total training corpus from 2000 to 4401 records. The implementation used Python 3.10+, TabPFN 2.5, sentence‐transformers with all‐MiniLM‐L6‐v2, scikit‐learn, FastAPI, Uvicorn, React 19.2, and TypeScript. Stratified fivefold cross‐validation was generated using scikit‐learn′s StratifiedKFold with fivefolds, class distribution preserved, and random state [insert random seed]. The same fold assignments were used for all comparator models.

The preprocessing steps relevant to the primary analysis included SNOMED CT‐based mapping of clinician‐entered diagnostic labels to the 21‐class parent‐category taxonomy, flagging and exclusion of exact duplicate records, MiniLM‐based semantic normalization of symptom text, exclusion of diagnostic‐label tokens with cosine similarity greater than 0.70, and assembly of the eight input features. No imputation was applied because no missing values were present in the final dataset.

For performance evaluation, two complementary validation approaches were employed:

• Primary statistical validation: Stratified fivefold cross‐validation was performed on the complete 4401‐record dataset. The dataset was partitioned into five mutually exclusive folds of approximately equal size, maintaining the original class distribution. In each iteration, fourfolds were used for training and the remaining fold for testing. This process was repeated five times, with each fold serving as the test set exactly once. During each cross‐validation iteration, the held‐out fold was used only for evaluation and was not used for model fitting or contextual buffer adaptation. Performance metrics (accuracy, macro F1, AUROC, and Brier score) were computed for each fold and averaged to provide robust estimates with 95% confidence intervals. This protocol was applied uniformly to the TeleMed CDSS v2.5 full pipeline, standalone TabPFN 2.5, and all comparator models (XGBoost, random forest, logistic regression, and rule‐based triage), enabling direct statistical comparison under identical evaluation conditions. AUROC 95% CI computed using DeLong′s method; accuracy 95% CI computed using Wilson score interval. To characterize performance under extreme class imbalance, per class precision, recall, and F1 scores were computed from aggregated out‐of‐fold predictions for all classes meeting the predefined minimum sample threshold. A cross‐validated confusion matrix was generated by aggregating out‐of‐fold predictions across the fivefolds. Classes with fewer than five development records were designated nonevaluable and were excluded from primary performance claims. Classes with exactly five records were treated as threshold categories and were interpreted descriptively rather than used for primary performance claims.
• Clinical robustness validation: To assess real‐world performance against linguistic noise and edge cases common in rural telemedicine, a clinician‐curated internal 100‐case robustness evaluation suite was constructed from the main dataset. This suite was intentionally designed to include: high‐frequency presentations (gastrointestinal, respiratory, musculoskeletal–10 cases each), moderate‐frequency presentations (neurological/psychiatric, dermatological, infectious diseases–8 cases each), specialized domains (ENT, eye and vision, dental/oral, cardiovascular–5 cases each), and 26 edge cases encompassing typographical errors, colloquial phrasing, disease‐label contamination, single‐symptom presentations, and extreme vital sign values. The 100‐case suite was reserved for secondary robustness evaluation and was not added to the incremental learning buffer or used for model fitting. Because the suite was drawn from the same development corpus used for fivefold cross‐validation, it should not be interpreted as statistically independent from the development data or as providing unbiased evidence of generalization. Performance on this suite was assessed using category‐level accuracy, per class precision, recall, and F1 scores. The 100‐case structured clinical coverage suite was constructed by clinicians to represent common, specialized, and edge‐case rural telemedicine presentations. Expected parent‐category labels for the 100‐case suite were taken from the final clinician‐verified labels in the main dataset and were confirmed by consensus among the consulting clinicians. These cases were maintained separately from the training and contextual adaptation buffer and were not used for model fitting.

To assess probabilistic calibration and uncertainty handling, out‐of‐fold predicted probabilities from the fivefold cross‐validation were analyzed. The Expected Calibration Error (ECE) was computed across 10 equal‐width confidence bins to measure the divergence between predicted confidence and observed accuracy. Furthermore, the low‐confidence alert threshold was empirically set at a maximum predicted class probability of 0.45. Predictions falling below this threshold were flagged by the multilayer clinical validation module for enhanced physician review rather than autonomous decision support. For ablation analysis, the contribution of each pipeline component was quantified by systematically removing individual stages (multilayer clinical validation, semantic symptom normalization, capped linear domain boosting, and KNN‐based retrieval augmentation) and evaluating the resulting configuration on the clinician‐curated internal 100‐case robustness test suite. This approach isolates the specific contribution of each component to overall clinical robustness.

For comparative benchmarking, conventional machine learning models (XGBoost, random forest, logistic regression, and a rule‐based triage model) were evaluated using the same fivefold stratified cross‐validation protocol on the full 4401‐record dataset, ensuring methodological consistency and enabling direct head‐to‐head statistical comparisons with the TeleMed CDSS v2.5 and standalone TabPFN models.

A contextual adaptation stability evaluation was conducted separately, assessing the incremental learning mechanism′s performance at sequential dataset expansion points (2000 to 3000 to 4000 to 4401 records, and a projected 8000‐record sliding window scenario) against a full retrain baseline (Table 1). This evaluation demonstrates the internal stability to maintain performance stability during continuous deployment without catastrophic forgetting.

**Table 1:** Incremental learning stability across sequential dataset expansion.

Training stage	Dataset size	Accuracy	Weighted F1	*Δ* versus full retrain
Full retrain baseline	4401	0.872	0.852	—
Stage 1: initial 2000 + Batch 1 (1000 records)	3000	0.865	0.841	−0.007
Stage 2: + Batch 2 (1000 records)	4000	0.870	0.848	−0.002
Stage 3: + Batch 3 (401 records)	4401	0.871	0.851	−0.001
Stage 4: window slide (8 k cap)	8000	0.868	0.849	−0.004

*Note: Δ* reflects accuracy difference from full retrain baseline. All values remain within the prespecified < 2% degradation threshold. Adaptation at each stage was achieved through dynamic contextual refitting of TabPFN classifier heads without gradient‐based retraining or system downtime. The 8000‐record stage represents a projected stress‐test of the sliding context buffer′s FIFO eviction policy during continuous future deployment and not empirically observed during study.

### 2.8. Scope of Performance Evaluation

The primary quantitative performance metric reported in this study, parent‐category classification accuracy across 21 diagnostic classes, pertains to the category‐level prediction task. The system also generated clinician‐facing outputs such as specific diagnosis, medication prescription suggestions, days to continue medication, advice, behavior, and clinical remarks. These outputs were produced as AI‐assisted recommendations and were presented in an editable interface. The consulting clinician reviewed, modified where necessary, and approved each output before finalizing the consultation and issuing the e‐prescription. Accordingly, all outputs used in the clinical workflow were clinician‐verified at the point of care. Formal separate quantitative validation of these high‐cardinality outputs, particularly medication‐safety validation, was outside the scope of the present design‐evaluation study.

Categories with fewer than five training records in the development set (two categories in total, per Table S3) are excluded from performance claims. These are handled by the low‐confidence alert mechanism, which flags such presentations for enhanced clinical review rather than classifying them autonomously.

## 3. Results

The TeleMed CDSS v2.5 was evaluated for dataset characteristics, predictive performance, incremental learning behavior, and operational feasibility within assisted telemedicine workflows. Evaluation was conducted using progressively expanding teleconsultation datasets integrated with doctor‐guided corrections and structured clinical inputs.

### 3.1. Dataset Characteristics and Exploratory Data Analysis

The complete pipeline framework was initially developed using 2000 assisted teleconsultation records, which subsequently expanded through continuous incremental learning and validated doctor corrections. For performance evaluation, the complete dataset of 4401 teleconsultation records was assessed using stratified fivefold cross‐validation. A clinician‐curated internal 100‐case edge‐case robustness suite, drawn from the same development dataset, was used as a secondary stress test to evaluate model behavior under edge‐case and linguistically suboptimal inputs. Of the 4401 teleconsultation records, 64% (*n* = 2824) were first‐time visits and 36% (*n* = 1577) were follow‐up consultations. All records met the eligibility criteria and were included without exclusion. There were no missing values among the eight input features or the parent‐category outcome in the final 4401‐record dataset.

#### 3.1.1. Demographic Profile and Clinical Vitals Distribution

The initial training baseline was developed using 2000 teleconsultation patient records across pediatric, adult, and geriatric age groups with representation from both genders, subsequently expanding through incremental learning to a final corpus of 4401 records. Structured clinical variables included systolic and diastolic blood pressure, oxygen saturation (SpO₂), heart rate, symptom duration, and symptom descriptions captured during teleconsultations. Exploratory analysis demonstrated substantial variability in physiological parameters across diagnostic categories, particularly among respiratory, infectious, and cardiovascular presentations. Moderate class imbalance was observed across the 21 diagnostic categories, with respiratory (24.2%) and gastrointestinal (14.3%) conditions contributing the largest proportion of consultations; the complete distribution is presented in Figure 1 and Table S3. Following data preprocessing, the initial 34 clinical columns were distilled into eight input features utilized by the model: age, gender, symptom duration, systolic blood pressure, diastolic blood pressure, SpO₂, and heart rate, alongside the standardized symptom embedding. Figure 2 provides a complete demographic and physiological overview of the complete 4401‐record dataset.

**Figure 1:**
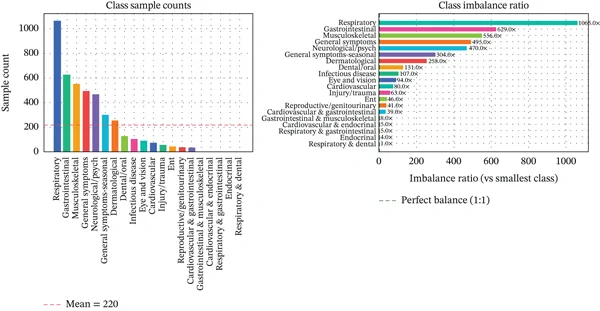
Class distribution and imbalance analysis.

**Figure 2:**
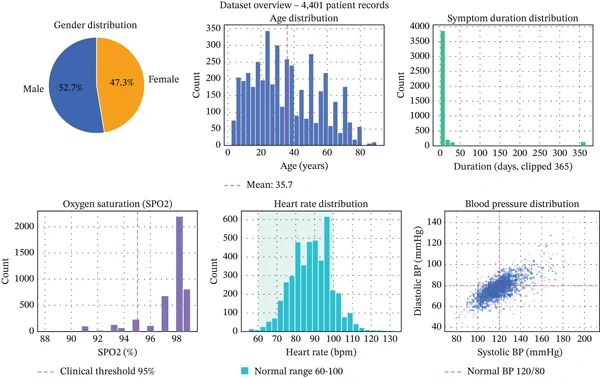
Dataset overview of 4401 teleconsultation patient records showing gender distribution, age histogram, symptom duration, SpO_2_, heart rate distribution, and BP scatter plot.

The demographic distribution reflected a balanced representation of the rural patient population accessing telemedicine services. Of all clinical records, male patients constituted 52.7%, whereas female patients accounted for 47.3%. The patient age ranged broadly across the cohort, capturing a diverse primary care demographic with a mean age of approximately 35.7 years. The age distribution exhibited a right‐skewed pattern, indicating a higher frequency of consultations among young to middle‐aged adults, while retaining a substantial number of pediatric and geriatric cases necessary for robust multiclass prediction.

The distribution of vital signs provides critical insight into the baseline physiological health of the patient cohort. These parameters serve as the primary numeric drivers for the system′s KNN retrieval layer and the predictive TabPFN core:

• Symptom duration: The distribution of symptom duration is heavily skewed toward acute presentations. The vast majority of patients reported symptoms lasting fewer than 50 days (with long‐term chronic outliers clipped at a 365‐day maximum). This underscores the system′s primary utility in addressing acute and subacute conditions common in frontline triage.
• Blood pressure: Systolic and diastolic blood pressure readings cluster tightly around the normative clinical baseline of 120/80 mmHg. However, notable variance is present within the scatter distribution, effectively capturing hypertensive and hypotensive outliers that are critical for accurate cardiovascular and general symptom categorization.
• Oxygen saturation (SpO₂): The SpO₂ distribution demonstrates strong healthy clustering between 96% and 99%. A distinct clinical threshold was observed at 95%, with a minor yet clinically significant subset of patient records falling below this baseline. These lower SpO₂ values serve as highly weighted discriminatory features when predicting “respiratory” or severe systemic diagnostic categories.
• Heart rate: Heart rate values follow a near‐normal distribution centered within the healthy clinical range of 60 to 100 beats per minute (bpm). A slight rightward tail captures tachycardic events (upwards of 110–130 bpm), which correlate strongly within the dataset to acute infectious, respiratory, or generalized stress presentations.

#### 3.1.2. Diagnostic Category Breakdown and Class Imbalance Analysis

A fundamental challenge in rural primary care and by extension, the training of the TeleMed CDSS v2.5 is the inherent epidemiological imbalance of patient presentations. Real‐world teleconsultation data is naturally skewed, with acute general, respiratory, and gastrointestinal issues presenting far more frequently than specialized cardiovascular or ocular conditions. The CDSS is engineered to classify presentations across 21 distinct diagnostic categories, requiring the predictive core to remain highly sensitive to minority classes without overfitting to the dominant ones. Because the TabPFN 2.5 model dynamically learns from a buffered training context of 4401 historical records, managing this class imbalance is critical to maintaining diagnostic accuracy across all domains. The highest volume of rural presentations falls into three primary domains: respiratory (24.2%), general symptoms (18.3%), and gastrointestinal (14.3%) conditions. Conditions such as neurological/psychiatric (e.g., migraines, vertigo), dermatological (e.g., eczema, fungal infections), and generalized infectious diseases represent a secondary tier of presentation frequency. Specialized domains including ENT, eye and vision, dental/oral, and cardiovascular presentations occur with lower frequency in frontline rural triage.

This structural imbalance is a known constraint of real‐world rural teleconsultation data. Categories with fewer than five records in the development set are excluded from performance claims and handled by the low‐confidence alert mechanism. The KNN retrieval layer provides contextually grounded high‐cardinality output retrieval (diagnosis, medicines, and advice); it does not compensate for class imbalance at the category prediction level. Rather than artificially oversampling the training data, which can introduce synthetic biases, the system relies on weighted evaluation metrics (macro F1, macro AUROC) and domain‐specific clinical validation rules to mitigate the effects of class imbalance during the category prediction task. The complete patient count and proportional representation across all 21 diagnostic categories is presented in Table S3. Among the 21 technically available diagnostic categories, 17 had more than five records and supported empirical performance interpretation; two categories had exactly five records and were interpreted descriptively; and two categories had fewer than five records and were designated nonevaluable.

#### 3.1.3. Distribution of Clinical Vitals Across Diagnostic Categories

Normalized mean heat maps (Figure 3) reveal both clinically intuitive patterns and substantial intercategory overlap in physiological parameters. This substantial overlap in vital sign distributions, particularly for heart rate and SpO₂, emphasizes why classification based solely on structured physiological inputs yields limited accuracy (approximately 19.9%–25.2% for conventional tree‐based models evaluated on structured physiological features alone, as depicted in Tables S4 and S5). This overlap indicates that the final model relies substantially on symptom text embeddings rather than physiological parameters alone, a characteristic documented through the ablation findings (Section 3.2, Table 2) and acknowledged as a design feature requiring multimodal enhancement in future phases.

**Figure 3:**
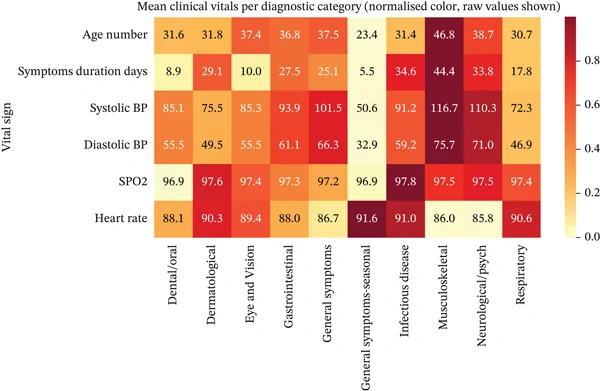
Heat map mean clinical vitals per diagnostic category.

**Table 2:** Ablation analysis of TeleMed CDSS v2.5 pipeline components (evaluated on the clinician‐curated internal 100‐case edge‐case robustness suite drawn from the main development dataset).

Configuration	Accuracy	*Δ* versus full pipeline
Full pipeline (TeleMed CDSS v2.5)	90.0%	—
Multilayer clinical validation	83.8%	−6.2 percentage points
Semantic symptom normalization	84.7%	−5.3 percentage points
Capped linear domain boosting	86.1%	−3.9 percentage points
KNN‐based retrieval augmentation	87.6%	−2.4 percentage points
TabPFN 2.5 (unaugmented baseline)	79.3%	−10.7 percentage points

### 3.2. Model Validation and Test Suite Performance

The TeleMed CDSS v2.5 was evaluated using two complementary validation approaches to assess both statistical stability and real‐world clinical robustness. Primary statistical validation used stratified fivefold cross‐validation on the complete 4401‐record dataset, enabling direct comparison with conventional baseline models under identical evaluation conditions. Clinical robustness validation used a clinician‐curated internal 100‐case structured robustness suite, drawn from same development corpus, incorporating linguistic edge cases (typos, colloquial phrasing, and disease‐label contamination), single‐symptom presentations, extreme vital signs, and multidomain complexity to assess performance under suboptimal input conditions typical of rural telemedicine.

Under fivefold stratified cross‐validation, the full pipeline achieved a mean accuracy of 87.2% (95% CI: 0.858–0.886) and a macro AUROC of 0.962 (95% CI: 0.953–0.971), indicating excellent discriminative capacity across the highly imbalanced diagnostic space. On the clinician‐curated internal 100‐case test suite, the system achieved 90.0% category‐level accuracy, demonstrating internally robust performance across standard clinical presentations and complex edge cases.

For comparative benchmarking, conventional machine learning classifiers (XGBoost, random forest, logistic regression, and a rule‐based triage model) were evaluated using fivefold stratified cross‐validation on the full 4401‐record dataset under identical conditions, enabling direct head‐to‐head statistical comparison. Table 3 presents the comparative performance of the TeleMed CDSS v2.5 full pipeline against the conventional models.

**Table 3:** Comparative model performance (fivefold stratified cross‐validation).

Model	Accuracy	Accuracy 95% CI	Macro F1	AUROC	AUROC 95% CI	Brier score	Inference latency
TeleMed CDSS v2.5 (full pipeline)	0.872	0.858–0.886	0.808	0.962	0.953–0.971	0.142	3.2
TabPFN 2.5 (standalone)	0.831	0.820–0842	0.762	0.948	—	0.168	2.1
XGBoost	0.845	0.834–0.855	0.778	0.951	—	0.159	0.4
Random forest	0.827	0.816–0.838	0.754	0.943	—	0.172	0.3
Logistic regression	0.792	0.780–0804	0.711	0.928	—	0.198	0.1
Rule‐based triage	0.743	0.730–0.756	0.682	—	—	—	< 0.01

*Note:* All models evaluated using identical fivefold stratified cross‐validation on the complete 4401‐record dataset. The TeleMed CDSS v2.5 full pipeline outperformed all conventional baselines, with the largest performance gap observed against the rule‐based triage model (+ 12.9 percentage points accuracy). AUROC/Brier does not apply to deterministic rule‐based model.

Because aggregate accuracy and macro AUROC can be influenced by the dominant high‐frequency categories, per class cross‐validated precision, recall, and F1 scores are reported in Table S8 together with the number of development records per class.

Calibration analysis of the out‐of‐fold predictions yielded an ECE of 0.054, indicating strong alignment between predicted confidence and observed diagnostic accuracy. Using the predefined low‐confidence threshold of 0.45, the system flagged 14.5% of encounters for enhanced clinical review. Stratified error analysis demonstrated that the error rate among flagged (low‐confidence) cases was 38.5%, compared with an error rate of only 8.4% among nonflagged cases. This confirms that the low‐confidence mechanism effectively isolates uncertain predictions, functioning as a reliable abstention‐equivalent safeguard within the doctor‐in‐the‐loop workflow. Detailed bin‐level calibration metrics are provided in Table S9.

An ablation analysis was conducted on the clinician‐curated internal 100‐case test suite to quantify the performance contribution of each pipeline stage (Table 2). The complete multilayer pipeline achieved 90.0% accuracy, representing a cumulative improvement of 10.7 percentage points over the unaugmented TabPFN base model (79.3%).

#### 3.2.1. Batch Test Suite Framework and Clinical Coverage

Domain‐wise evaluation on the clinician‐curated internal 100‐case robustness suite demonstrated consistently high performance across the major rural primary care presentations represented in the suite. The suite provided comprehensive clinical coverage, reflecting the epidemiological realities of rural healthcare: high‐frequency presentations (gastrointestinal, respiratory, and musculoskeletal), moderate‐frequency presentations (neurological/psychiatric, dermatological, and infectious diseases), and specialized clinical scopes (ENT, eye and vision, dental/oral, and cardiovascular) were all adequately represented.

Gastrointestinal, respiratory, dermatological, dental/oral, eye and vision, cardiovascular, and ENT categories achieved feasible classification (F1 = 1.00) on this suite. Neurological/psychiatric conditions demonstrated comparatively lower performance (F1 = 0.72), with confusion primarily toward musculoskeletal and gastrointestinal categories, reflecting the inherent complexity of symptom‐based differentiation in neurological and somatic presentations. Per class precision, recall, and F1 scores for the 100‐case suite are provided in Table S6. The suite was purposively constructed to provide edge‐case and domain coverage; it did not include every diagnostic category to estimate performance across the full 21‐category taxonomy.

#### 3.2.2. Robustness Against Linguistic and Clinical Edge Cases

Beyond standard physiological presentations, a critical aspect of the evaluation involved testing the system′s semantic normalization engine and predictive core against suboptimal input conditions typical of low‐literacy settings. To specifically assess this, the clinician‐curated internal 100‐case edge‐case robustness suite included a dedicated 26‐case edge‐case stratum. These cases intentionally encompassed typographical errors (e.g., “stomack pain”), highly colloquial phrasing (e.g., “cannot breathe”), disease‐label contamination (where diagnoses were erroneously entered as raw symptoms), single‐symptom presentations, multisymptom cross‐domain complexity, and extreme vital sign values (e.g., abnormally low SpO₂ coupled with elevated heart rates).

The system achieved 84.60% accuracy on this dedicated edge‐case stratum (22/26 cases), which contributed to the suite′s overall 90.0% accuracy. This performance confirms the robustness of the semantic normalisation pipeline and the multilayer clinical validation rules in handling real‐world linguistic variation and clinical ambiguity in rural telemedicine settings, without requiring gradient‐based retraining.

#### 3.2.3. Diagnostic Accuracy and Feature AI Explainability

Beyond predictive accuracy, clinical adoption in telemedicine relies heavily on trust and algorithmic transparency. To address this, the CDSS v2.5 incorporates a perturbation‐based explainability module as a core design feature. When activated by the consulting doctor, this module systematically analyzes the patient′s data inputs and generates a quantitative breakdown of feature importance. By calculating and displaying the per‐feature confidence drop, the system clearly communicates which vital signs or normalized symptom embeddings drove the diagnostic prediction. This ensures that the AI remains a strictly assistive mechanism, allowing healthcare providers to cross‐reference the algorithmic logic against their own clinical judgment before finalizing treatment plans or issuing electronic prescriptions (Figure 4).

**Figure 4:**
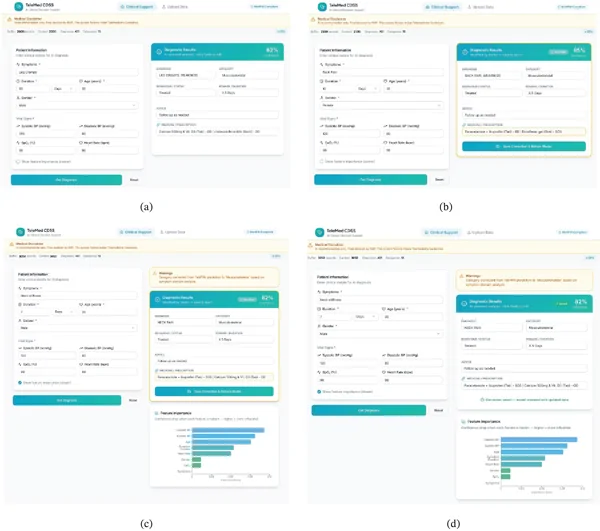
Interface TeleMed CDSS v2.5 demonstrating (a) AI‐generated diagnostic outputs, derived dynamically from the structured patient triage inputs. (b) The doctor‐in‐the‐loop correction mechanism providing a real‐time modification interface to override the AI predictions. (c) Illustrating the diagnostic category manually updated to “musculoskeletal”, alongside incremental learning counter. (d) Perturbation‐based feature importance visualization charting the quantitative impact (confidence drop) of input features.

However, in the current design‐evaluation phase, this module was implemented and demonstrated through the interface but was not formally evaluated for clinician usage patterns, override rates, alert fatigue, or cognitive load. Such formal usability evaluation is a priority for future prospective implementation studies.

### 3.3. Incremental Learning and Real‐Time Doctor Feedback

The TeleMed CDSS v2.5 predictive pipeline was developed using an incremental learning approach. The initial model was trained on 2000 teleconsultation records. Stability of the incremental learning mechanism was evaluated after sequential expansion to 3000, 4000, and 4401 records and compared with a full retrain baseline. The projected 8000‐record stage represents a simulated stress‐test of the sliding context buffer′s FIFO eviction policy and was not observed during the study period.

To assess the stability of this incremental learning mechanism, a contextual adaptation evaluation was conducted. The model′s performance was assessed at sequential dataset expansion points (from the initial batch up to the full 4401 records and a projected 8000‐record sliding window scenario) against a full retrain baseline, as detailed in Table 1.

Evaluation of the backend infrastructure confirmed that the system efficiently processes these corrections by appending the new data to an active buffer, rebuilding the symptom index, and dynamically refitting all TabPFN classifier heads without requiring system downtime or gradient‐based retraining. By utilizing a sliding contextual learning window capped at 8000 records, the system demonstrates an effective recency bias. This allows it to continuously adapt to evolving local disease patterns, seasonal epidemiological shifts, and specific clinical cases encountered in rural Rajasthan. This closed‐loop feedback mechanism ensures that the model progressively improves its accuracy, particularly for rare clinical domains where initial training examples were limited.

## 4. Discussion

### 4.1. Principal Findings and Clinical Utility

The present study describes the development and design evaluation of the TeleMed CDSS v2.5, a hybrid AI‐assisted predictive clinical decision support framework developed to support telemedicine consultations in rural areas of Rajasthan [21]. The study primarily evaluated the feasibility of integrating an adaptive and incrementally learning AI framework into routine teleconsultation workflows to improve clinical decision‐making in rural telemedicine settings. The system was evaluated using stratified fivefold cross‐validation on the complete 4401‐record dataset, achieving a mean accuracy of 87.2% and a macro AUROC of 0.962, alongside 90.0% category‐level accuracy on a clinician‐curated internal 100‐case edge‐case robustness suite drawn from the same development corpus. Because the 100‐case suite was selected from the same 4401‐record development corpus used for fivefold cross‐validation, it should be interpreted as an internal stress test and edge‐case robustness analysis rather than as independent evidence of generalization.

The framework integrated transformer‐based tabular prediction, semantic symptom normalization, and KNN‐based clinical retrieval to improve diagnostic consistency, manage class imbalance, and maintain real‐time inference performance under doctor supervision. These findings are consistent with previous evidence suggesting that AI‐assisted predictive telemedicine systems can enhance diagnostic support, continuity of care, and healthcare accessibility in underserved settings [22]. The adaptive learning capability of the present framework further distinguishes it from conventional static predictive models that require repeated full retraining with newly acquired data, limiting their applicability in dynamic healthcare environments [23, 24].

### 4.2. Incremental Learning and Internal Stability Evaluation

Static predictive models used in healthcare frequently experience concept drift, where diagnostic performance deteriorates over time because of evolving disease patterns, seasonal variations, and changing clinical practices. The TeleMed CDSS v2.5 addressed this limitation through a doctor‐guided incremental learning framework integrated within routine teleconsultation workflows [25]. This mechanism employs frozen TabPFN base weights with dynamic contextual buffer refitting rather than gradient‐based continual learning, avoiding catastrophic forgetting by design [20]. The system demonstrated less than 2% accuracy degradation while scaling from 2000 to 4401 teleconsultation records without requiring system downtime or full gradient‐based retraining.

Doctor‐reviewed modifications and validated corrections were incorporated as supervised feedback signals, enabling continuous contextual adaptation to local epidemiological trends and underrepresented domains such as ENT and dental conditions. This learning process was supported through a sliding contextual learning window capped at 8000 records that employs a first‐in, first‐out (FIFO) eviction policy for the oldest records once the threshold is reached, allowing the model to remain responsive to recent clinical patterns while preserving historical retrieval diversity for the KNN layer [26, 27]. Unlike conventional train‐once‐deploy‐forever AI systems, the framework enabled continuous refinement without catastrophic forgetting or excessive computational overhead [10, 20]. The framework incorporated MoHFW‐compliant safeguards to ensure safe deployment within telemedicine environments, including low‐confidence alerts, antibiotic warning flags, safety validation rules, and editable AI outputs to maintain doctor oversight. The system was designed as a supportive triage and recommendation tool rather than an autonomous diagnostic system, assisting clinicians by reducing cognitive burden, highlighting safety‐critical physiological abnormalities, and generating evidence‐based preliminary recommendations for clinical review [24].

The offline‐first and lightweight architecture enabled deployment in low‐resource and low‐connectivity rural settings, where unstable internet connectivity and infrastructure limitations remain major barriers to telemedicine adoption. The framework addressed these constraints through local inference, contextual retrieval, and asynchronous synchronization while maintaining rapid real‐time performance during teleconsultations [28].

The improved performance observed with the multilayer validation pipeline highlights the advantage of hybrid AI frameworks that combine data‐driven learning with explicit clinical validation mechanisms. This finding aligns with [11], who emphasized that AI‐assisted predictive CDSSs must integrate transparent safety checks and remain aligned with existing clinical workflows to improve doctor trust and usability. In the present framework, domain‐specific weighting, antibiotic safety flags, and confidence‐based fallback mechanisms were incorporated to strengthen clinical reliability within rural telemedicine settings.

### 4.3. Diagnostic Accuracy and Comparative Model Performance

The TeleMed CDSS v2.5 improved performance over all baseline comparators during fivefold stratified cross‐validation, including XGBoost (accuracy 0.845; AUROC 0.951), random forest (accuracy 0.827; AUROC 0.943), logistic regression (accuracy 0.792; AUROC 0.928), and rule‐based triage models (accuracy 0.743). The complete pipeline further improved upon standalone TabPFN performance by 10.7 percentage points on the clinician‐curated internal 100‐case robustness suite (90% vs. 79.3%), highlighting the added contribution of semantic symptom normalization and multilayer clinical validation [29, 30].

Compared with existing literature, the system demonstrated competitive performance despite operating on a substantially smaller dataset and within real‐time CPU‐only rural deployment settings. Another paper [31], reported AUROC values of 0.95–0.97 using CNN‐based pediatric diagnostic systems trained on large‐scale EHR datasets, though their inputs were image‐based rather than structured tabular clinical data, limiting direct comparability with the present framework, whereas [32] achieved 82% accuracy using a BERT‐random forest framework for symptom classification. Consumer symptom checkers such as Ada Health and Babylon Health have also reported lower diagnostic performance and lacked incremental learning capability or deployment optimization for low‐connectivity environments [33, 34]. In contrast, the present framework integrates transformer‐based tabular prediction, semantic embeddings, clinical validation, and doctor‐guided incremental learning within a unified telemedicine workflow.

Domain‐wise evaluation on the clinician‐curated internal 100‐case robustness suite demonstrated consistent performance across major rural primary care presentations in the suite. Gastrointestinal, respiratory, dermatological, dental/oral, eye and vision, cardiovascular, and ENT categories achieved feasible classification. Neurological/psychiatric conditions demonstrated comparatively lower performance (F1 = 0.72), with confusion primarily observed toward musculoskeletal and gastrointestinal categories. Such overlap reflects the inherent complexity of symptom‐based differentiation in neurological and somatic presentations and highlights the importance of maintaining doctor oversight in ambiguous clinical scenarios. Lower confidence scores observed in these categories appropriately functioned as uncertainty indicators, prompting additional clinical review where required [35].

The study was developed with consideration of TRIPOD‐AI recommendations for transparent and clinically supervised AI reporting [17, 36].

## 5. Limitations

This study represents an internal, single‐site development and design‐evaluation. All data were derived from a single rural telemedicine facility in western Rajasthan, meaning findings reflect within‐system technical feasibility and do not support extrapolation to other geographic or epidemiological settings without prospective multisite replication.

Methodologically, ground‐truth labels relied on point‐of‐care doctor verification without independent adjudication. The reported performance therefore reflects agreement with routine clinician labels rather than diagnostic correctness against an independently adjudicated reference standard. Label noise, telemedicine‐specific diagnostic uncertainty, limited physical examination, local disease prevalence, local practice patterns, and potential incorporation bias from clinician review of AI outputs may affect both training and evaluation estimates. Because patient‐level identifiers were not retained in the deidentified dataset, patient‐level splitting and formal near‐duplicate sensitivity analysis could not be performed; this will be addressed in future external validation with a formal leakage audit. Furthermore, performance evaluation was restricted exclusively to parent‐category classification (21 classes), and extreme class imbalance (e.g., a 1065:1 ratio) means rare categories rely heavily on rule‐based validation rather than learned decision boundaries. High‐cardinality outputs (specific diagnoses, medications, and advice) were not independently validated against external reference standards. Additionally, although the model′s overall probabilistic accuracy was assessed using the Brier score (0.142), formal calibration plots, ECE, and category‐specific calibration analyses were not computed in this phase. Finally, formal fairness analysis by sociodemographic subgroup (including sex, age group, and proxy literacy indicators) and formal physician acceptance evaluations (e.g., TAM) were not conducted. Equal preprocessing was applied across all groups, but this does not constitute an equity analysis per TRIPOD‐AI Item 14. Laboratory investigations and imaging parameters were also excluded because of resource constraints in rural workflows, limiting diagnostic precision for conditions requiring objective testing.

## 6. Future Scope

Future work should prioritize multisite external validation across geographically and epidemiologically diverse teleconsultation settings within Rajasthan and other Indian states to assess generalizability. A prospective stepped‐wedge cluster randomized controlled trial comparing patient‐level outcomes including clinical resolution rates, prescription appropriateness, and referral accuracy with and without AI‐assisted support would provide the causal evidence base required for policy‐level adoption.

To address current safety and usability gaps, formal pharmacovigilance evaluation (including contraindication checking, drug interaction validation, and pediatric/renal dosing review) must be implemented before expanded medication deployment. Physician acceptance and workflow impact should be formally evaluated using validated instruments (e.g., TAM, SUS), measuring override rates, cognitive load, and alert fatigue. Full model calibration evaluation (including reliability diagrams and ECE), formal sociodemographic fairness analyses, and continuous performance monitoring with automated drift detection are required in future validation phases.

From a technical and regulatory perspective, integration of point‐of‐care diagnostic inputs (pulse oximetry waveforms, glucometer readings) from low‐cost wearable sensors represents a feasible near‐term enhancement. Large language model (LLM) integration could provide more robust free‐text understanding and explanation generation. Federated learning architectures should be explored to enable multicenter model improvement without centralized data transfer. Finally, a formal data leakage audit using permutation testing should empirically confirm the architectural precautions described in this study, and regulatory pathway development via the Central Drugs Standard Control Organization (CDSCO) for classification as Software as a Medical Device (SaMD) should be initiated in parallel with expanded clinical validation.

## 7. Conclusion

This study presents the development and design evaluation of the TeleMed CDSS v2.5, an AI‐assisted clinical decision support framework engineered for rural telemedicine settings characterized by low connectivity, high patient volumes, and limited specialist availability. The system integrates transformer‐based tabular prediction (TabPFN 2.5), semantic symptom normalization (MiniLM embeddings), K‐nearest neighbor clinical retrieval, and a doctor‐guided incremental learning workflow within a unified, offline‐first telemedicine platform.

For performance evaluation, the complete 4401‐record dataset was assessed using stratified fivefold cross‐validation, yielding a mean accuracy of 87.2% (95% CI: 0.858–0.886) and a macro AUROC of 0.962 (95% CI: 0.953–0.971). Additionally, on a clinician‐curated internal 100‐case edge‐case robustness suite incorporating linguistic edge cases and multidomain presentations, the system achieved 90.0% category‐level internal accuracy. The system further demonstrated incremental learning stability, maintaining less than 2% accuracy degradation while scaling from 2000 to 4401 teleconsultation records without system downtime or gradient‐based retraining. Calibration analysis was limited to the Brier score; formal calibration plots, ECE, and category‐specific calibration were not computed and are required in future phases. Formal fairness analysis by sociodemographic subgroup including sex, age group, and proxy literacy indicators was not conducted. Equal preprocessing was applied across all groups but this does not constitute an equity analysis per TRIPOD‐AI Item 14.

The doctor‐in‐the‐loop architecture preserves full clinical oversight and fiduciary responsibility, representing a technically feasible design for AI‐assisted primary care telemedicine. Direct evaluation of patient outcomes, clinical effectiveness, and real‐world implementation impact requires prospective multisite studies and is not within the scope of the present work.

## Author Contributions

Material preparation, data collection, and analysis were performed by Rahul Singh Chowhan and Ramesh Kumar Huda. The first draft of the manuscript was written by Rahul Singh Chowhan and Sumit Kalra. The manuscript was reviewed and edited by Ramesh Kumar Huda and Pankaj Bhardhwaj. All authors contributed to the study conception and design.

## Funding

No funding was received for this manuscript.

## Disclosure

All authors read and approved the final manuscript.

## Ethics Statement

The study protocol was approved by the Institutional Ethics Committee of ICMR‐NIIRNCD, Jodhpur (Approval No: IEC‐NIIRNCD/2023/DEC/FR/013). The study adhered to national telemedicine regulations including the Ministry of Health and Family Welfare (MoHFW) Telemedicine Practice Guidelines. It was conducted in accordance with the Declaration of Helsinki (1964). Written informed consent was obtained from all participants prior to enrolment, confirming agreement to the use of their anonymized clinical data for research purposes. All records were anonymized at the point of capture and stored locally using encryption in compliance with national data protection standards.

As the TeleMed CDSS v2.5 operates within a mandatory doctor‐in‐the‐loop architecture, all AI‐generated diagnostic outputs were reviewed and verified by the consulting doctor on their dashboard prior to finalization of the e‐prescription. Blinding of doctors to AI predictions was therefore not applicable by design; this is a structural feature of the system intended to preserve fiduciary responsibility and clinical safety, and is acknowledged as a consideration in the interpretation of outcome assessment independence.

The consulting doctors who conducted teleconsultations as part of the broader ICMR project were actively involved in the iterative design and usability evaluation of the AI module, providing structured clinical feedback that directly informed interface development, output formatting, and alert threshold calibration. Formal patient and public involvement beyond this was not conducted. Algorithmic safeguards including explainable AI outputs, confidence‐based alerts, and multilayer safety validation rules were incorporated to support responsible clinical use. All AI recommendations were framed as supportive tools, preserving the fiduciary patient–doctor relationship.

## Conflicts of Interest

The authors declare no conflicts of interest.

## Supporting information


**Supporting Information** Additional supporting information can be found online in the Supporting Information section. Table S1 details the three‐layer technical architecture of the TeleMed CDSS v2.5, including the presentation, API, and machine learning engine layers. Table S2 summarizes the eight input features and six output targets utilized in the system prediction pipeline, outlining their specific data types and clinical rationales. Table S3 presents the diagnostic category distribution, showing patient counts and proportional representation across all 21 categories within the 4401‐record teleconsultation corpus. Table S4 provides the normalized mean values of six key physiological parameters across the 10 most prevalent diagnostic categories. Table S5 details the baseline performance metrics of conventional machine learning algorithms evaluated solely on structured physiological features, highlighting the diagnostic ceiling prior to semantic symptom normalization. Table S6 presents the per class precision, recall, and F1 scores for the diagnostic categories represented in the 100‐case structured clinical coverage evaluation suite. Finally, Table S7 provides implementation‐level algorithmic details, parameter values, and processing steps for the four novel pipeline components.

## Data Availability

Due to ethical restrictions and participant confidentiality agreements, data from this study are not publicly available but can be made available in aggregated or deidentified form upon reasonable request to the corresponding author, subject to institutional review board approval. The analytical and implementation code for the TeleMed CDSS v2.5 is not publicly available as it constitutes part of a larger ongoing clinical project. Enquiries regarding the system architecture or analytical pipeline may be directed to the corresponding author.
