# Supplementary material for: Design and Internal Validation of an AI‐Assisted Clinical Decision Support System (CDSS) for Telemedicine Services to Augment Patient Care in Rural Areas of Rajasthan
Source: Int J Telemed Appl. 2026 Oct 28;2026:8245320. doi: 10.1155/ijta/8245320 (PMC13716943; doi:10.1155/ijta/8245320)
Supplement: Supplementary file 1 [file IJTA-2026-8245320-s001.docx]

**Design and Evaluation of an AI-Assisted Clinical Decision Support System (CDSS) for Telemedicine Services to Augment Patient Care in Rural Areas of Rajasthan**

**Supplementary Tables**

Manuscript ID: 8245320

List of Supplementary Tables

- Supplementary Table S1. Three-layer technical architecture of the TeleMed CDSS v2.5
- Supplementary Table S2. Summary of Input Features and Output Targets in the TeleMed CDSS v2.5
- Supplementary Table S3. Diagnostic Category Distribution across the 4,401-Record Dataset
- Supplementary Table S4. Normalised Mean Clinical Vitals per Diagnostic Category
- Supplementary Table S5. Conventional Machine Learning Model Performance on Structured Physiological Features Alone
- Supplementary Table S6. Per-Class Precision, Recall, and F1 Scores across Diagnostic Categories in the Clinician-Curated Internal 100-Case Robustness Suite
- Supplementary Table S7. Algorithmic Pseudocode for Novel TeleMed CDSS v2.5 Pipeline Components
- Supplementary Table S8. Cross-validated per-class performance for parent-category classification
- Supplementary Table S9. Calibration and Low-Confidence Threshold Behaviour

**Supplementary Table S1. Three-Layer Technical Architecture of the TeleMed CDSS v2.5**

Three-layer system architecture of the TeleMed CDSS v2.5, covering the presentation interface, API layer, and machine learning engine, with the corresponding technologies and functional roles for each component.

| **Layer** | **Technology** | **Role** |
| --- | --- | --- |
| Presentation | React 19.2 + TypeScript + TailwindCSS + ShadCN UI | Two-page single-page application: Clinical Decision Support and Data Upload interfaces |
| API | FastAPI + Uvicorn (Python 3.10+) | REST endpoints, CORS handling, model loading, lifespan management |
| ML Engine | TabPFN 2.5 + MiniLM (all-MiniLM-L6-v2) + scikit-learn (Python 3.10) | Six-stage prediction pipeline: semantic symptom normalisation, category classification via Decimal Positioning, KNN retrieval, domain boosting, multi-layer clinical validation, and contextual adaptation |

**Supplementary Table S2. Summary of Input Features and Output Targets in the TeleMed CDSS v2.5**

Complete specification of all eight input features and six output targets used in the TeleMed CDSS v2.5 prediction pipeline, with data types, clinical rationale, and evaluation status for each variable.

| **Variable Type** | **Feature** | **Data Type** | **Clinical Rationale** | **Evaluation Status** |
| --- | --- | --- | --- | --- |
| Input | Age | Numeric (integer, years) | Fundamental risk stratifier across paediatric, adult, and geriatric groups | Model input |
| Input | Gender | Categorical (Male/Female) | Gender-specific disease prevalence patterns; contributes within multivariate context | Model input |
| Input | Symptoms | Free text → 384-dimensional embedding | Primary diagnostic signal; semantically normalised before prediction | Model input |
| Input | Symptom Duration (days) | Numeric (integer, days) | Differentiates acute, sub-acute, and chronic presentations | Model input |
| Input | Systolic BP (mmHg) | Numeric (integer) | Cardiovascular risk and haemodynamic status | Model input |
| Input | Diastolic BP (mmHg) | Numeric (integer) | Cardiovascular risk and haemodynamic status | Model input |
| Input | SpO₂ (%) | Numeric (integer, %) | Respiratory compromise indicator; clinically important threshold at 95% | Model input |
| Input | Heart Rate (bpm) | Numeric (integer, bpm) | Physiological stress and cardiovascular marker | Model input |
| Output | Parent Category | 21-class classification | Broad diagnostic domain; primary classification target | Primary quantitatively evaluated output; final category clinician-verified |
| Output | Diagnosis | Free text (451 unique values) | Specific diagnostic label retrieved through KNN semantic similarity search | Clinician-supervised output; reviewed, modified if necessary, and approved by the consulting doctor; not separately quantified using classification accuracy |
| Output | Behaviour | 3-class classification | Clinical disposition: Treated / Referred / Follow-up | Clinician-supervised output; reviewed and approved by the consulting doctor; not included in primary quantitative performance claims |
| Output | Advice | Free text (157 unique values) | Clinical management guidance retrieved through KNN similarity search | Clinician-supervised output; reviewed, modified if necessary, and approved by the consulting doctor; not separately quantified using classification accuracy |
| Output | Medicines | Structured prescription suggestion, including medication and days to continue medicine (2,842 unique medication-related values) | AI-assisted medication recommendation retrieved through KNN; presented for clinician review before e-prescription | Clinician-supervised medication output; reviewed, modified if necessary, and explicitly approved by the consulting doctor before prescription issuance; not separately quantified; formal medication-safety validation not performed |
| Output | Remark | 14-class classification | Clinical context annotation | Clinician-supervised output; reviewed by the consulting doctor; not included in primary quantitative performance claims |

***Note:*** *All outputs were AI-assisted outputs presented in an editable interface. The consulting clinician reviewed, modified where necessary, and approved these outputs before finalisation of the consultation and issuance of the e-prescription. These outputs were clinician-verified at the point of care but were not separately quantitatively evaluated as primary study endpoints. Mandatory physician approval and rule-based safety flags are design controls and do not substitute for formal medication-safety validation.*

**Supplementary Table S3. Diagnostic Category Distribution across the 4,401-Record Dataset**

Patient counts and proportional representation across all 21 diagnostic categories in the complete 4,401-record teleconsultation corpus. Categories are ordered by frequency.

| **Diagnostic Category** | **Patient Count (n)** | **Proportion (%)** | **Training Records Flag** |
| --- | --- | --- | --- |
| Respiratory | 1,065 | 24.2 | Included in performance claims |
| Gastrointestinal | 629 | 14.3 | Included in performance claims |
| Musculoskeletal | 556 | 12.6 | Included in performance claims |
| General Symptoms | 495 | 11.2 | Included in performance claims |
| Neurological / Psychiatric | 470 | 10.7 | Included in performance claims |
| General Symptoms — Seasonal | 304 | 6.9 | Included in performance claims |
| Dermatological | 258 | 5.9 | Included in performance claims |
| Dental / Oral | 131 | 3.0 | Included in performance claims |
| Infectious Disease | 107 | 2.4 | Included in performance claims |
| Eye and Vision | 94 | 2.1 | Included in performance claims |
| Cardiovascular | 80 | 1.8 | Included in performance claims |
| Injury / Trauma | 63 | 1.4 | Included in performance claims |
| ENT | 46 | 1.0 | Included in performance claims |
| Reproductive / Genitourinary | 41 | 0.9 | Included in performance claims |
| Cardiovascular & Gastrointestinal | 39 | 0.9 | Included in performance claims |
| Gastrointestinal & Musculoskeletal | 8 | 0.2 | Included in performance claims |
| Cardiovascular & Endocrinal | 5 | 0.1 | Threshold (reviewed case-by-case) |
| Respiratory & Gastrointestinal | 5 | 0.1 | Threshold (reviewed case-by-case) |
| Endocrinal | 4 | 0.1 | Excluded from accuracy claims; low-confidence alert triggered |
| Respiratory & Dental | 1 | 0.0 | Excluded from accuracy claims; low-confidence alert triggered |
| TOTAL | 4,401 | 100.0 |  |

*Note: The imbalance ratio between the most frequent (Respiratory, n = 1,065) and least frequent (Respiratory & Dental, n = 1) categories is approximately 1,065:1. Weighted evaluation metrics (macro F1, macro AUROC) were used throughout to account for this imbalance. Categories with fewer than five training records are excluded from performance claims per section 2.8 of the manuscript and are instead flagged by the low-confidence alert mechanism for enhanced clinical review.*

**Supplementary Table S4. Mean Clinical Vitals per Diagnostic Category**

Mean values of six key clinical parameters across the ten most prevalent diagnostic categories in the 4,401-record dataset. Values represent raw means. Elevated systolic and diastolic blood pressure in the Neurological/Psychiatric category and the overlap in heart rate and SpO₂ across several categories demonstrate the limited diagnostic sufficiency of physiological parameters alone, motivating the integration of semantic symptom normalisation in the TeleMed CDSS v2.5 pipeline.

| **Diagnostic Category** | **Mean Age (yrs)** | **Symptom Duration (days)** | **Systolic BP (mmHg)** | **Diastolic BP (mmHg)** | **SpO₂ (%)** | **Heart Rate (bpm)** |
| --- | --- | --- | --- | --- | --- | --- |
| Dental / Oral | 31.6 | 8.9 | 85.1 | 55.5 | 96.9 | 88.1 |
| Dermatological | 31.8 | 29.1 | 75.5 | 49.5 | 97.6 | 90.3 |
| Eye and Vision | 37.4 | 10.0 | 85.3 | 55.5 | 97.4 | 89.4 |
| Gastrointestinal | 36.8 | 27.5 | 93.9 | 61.1 | 97.3 | 88.0 |
| General Symptoms | 37.5 | 25.1 | 101.5 | 66.3 | 97.2 | 86.7 |
| Infectious Disease | 23.4 | 5.5 | 50.6 | 32.9 | 96.9 | 91.6 |
| Musculoskeletal | 31.4 | 34.6 | 91.2 | 59.2 | 97.8 | 91.0 |
| Neurological / Psychiatric | 46.8 | 44.4 | 116.7 | 75.7 | 97.5 | 86.0 |
| Respiratory | 38.7 | 33.8 | 110.3 | 71.0 | 97.5 | 85.8 |
| General Symptoms - Seasonal | 30.7 | 17.8 | 72.3 | 46.9 | 97.4 | 90.6 |

*Note: Values are raw mean values extracted from the 4,401-record dataset. The remaining 11 diagnostic categories are not shown due to small sample sizes.*

**Supplementary Table S5. Conventional Machine Learning Model Performance on Structured Physiological Features Alone**

Accuracy, macro F1, and weighted F1 for five conventional machine learning algorithms evaluated on an 80/20 stratified split using structured physiological features alone (top 15 diagnostic classes), prior to semantic symptom normalisation. All models performed in the range of 19.9%–25.2% accuracy, demonstrating the diagnostic ceiling imposed by vital-sign-only classification and justifying the integration of semantic symptom embeddings in the full pipeline.

| **Model** | **Accuracy** | **Macro F1** | **Weighted F1** |
| --- | --- | --- | --- |
| Gradient Boosting | 0.250 | 0.153 | 0.231 |
| Random Forest | 0.252 | 0.120 | 0.215 |
| KNN (k=5) | 0.199 | 0.111 | 0.195 |
| Logistic Regression | 0.243 | 0.086 | 0.155 |
| Majority Class Baseline | 0.243 | 0.026 | 0.095 |

*Note: Evaluation restricted to top 15 diagnostic classes to exclude categories with fewer than 5 samples in the test split. Features used: Age, Gender, Systolic BP, Diastolic BP, SpO₂, Heart Rate, Symptom Duration. Symptom text was excluded as it requires MiniLM embedding, which was not applied in this baseline evaluation. Note: This is a separate baseline evaluation conducted to demonstrate the insufficiency of physiological features alone; it is distinct from the primary validation framework (5-fold stratified cross-validation and 100-case prospective test suite) used for the full TeleMed CDSS v2.5 pipeline.*

**Supplementary Table S6. Per-Class Precision, Recall, and F1 Scores across Diagnostic Categories in the Clinician-Curated Internal 100-Case Robustness Suite**

Per-class precision, recall, and F1 scores for all diagnostic categories represented in the 100-case structured clinical coverage evaluation suite. Categories not represented in the suite (due to purposive domain-coverage design) are indicated as N/A. Categories with fewer than 5 training records are flagged as excluded from performance claims per section 2.8 of the manuscript.

| **Diagnostic Category** | **n in Suite** | **Precision** | **Recall** | **F1 Score** | **Performance Note** |
| --- | --- | --- | --- | --- | --- |
| Respiratory | 10 | 1.00 | 1.00 | 1.00 | Perfect classification (standard cases) |
| Gastrointestinal | 10 | 1.00 | 1.00 | 1.00 | Perfect classification |
| Musculoskeletal | 10 | 0.90 | 0.90 | 0.90 | Minor confusion with Neurological |
| Neurological / Psychiatric | 8 | 0.75 | 0.75 | 0.72 | Lowest performance; confusion with Musculoskeletal and Gastrointestinal |
| Dermatological | 8 | 1.00 | 1.00 | 1.00 | Perfect classification |
| Infectious Disease | 8 | 0.88 | 0.88 | 0.87 | Minor confusion with General Symptoms |
| ENT | 5 | 1.00 | 1.00 | 1.00 | Perfect classification |
| Eye and Vision | 5 | 1.00 | 1.00 | 1.00 | Perfect classification |
| Dental / Oral | 5 | 1.00 | 1.00 | 1.00 | Perfect classification |
| Cardiovascular | 5 | 1.00 | 1.00 | 1.00 | Perfect classification |
| Edge Cases (All Domains) | 26 | 0.85 | 0.85 | 0.84 | Includes typos, colloquialisms, single symptoms, extreme vitals, and contamination (22/26 correct) |
| General Symptoms | N/A | - | - | - | Not represented in the 100-case coverage suite |
| General Symptoms –Seasonal |  | - | - | - | Not represented in the 100-case coverage suite |
| Injury / Trauma |  | - | - | - | Not represented in the 100-case coverage suite |
| Reproductive / Genitourinary |  | - | - | - | Not represented in the 100-case coverage suite |
| Cardiovascular & Gastrointestinal |  | - | - | - | Not represented in the 100-case coverage suite |
| Endocrinal |  | - | - | - | <5 training records; excluded from claims. Low-confidence alert triggered. |
| Respiratory & Dental |  | - | - | - | <5 training records; excluded from claims. Low-confidence alert triggered. |
| Gastrointestinal & Musculoskeletal |  | - | - | - | Not represented in coverage suite |
| Cardiovascular & Endocrinal |  | - | - | - | Not represented in coverage suite |
| Respiratory & Gastrointestinal |  | - | - | - | Not represented in coverage suite |
| **TOTAL / MEAN** | **100** | **0.900** | **0.900** | **0.895** | **Macro average across represented categories** |

*F1 scores reflect macro-averaged per-class computation on the 100-case structured clinical coverage evaluation suite. The suite was purposively constructed; class representation reflects epidemiological coverage priorities rather than random sampling. Categories with N/A in the suite column were not included in the coverage evaluation due to insufficient training records (<5) or low prioritisation in the domain-coverage design. Overall accuracy of 90.0% (90/100 cases correct) reflects category-level classification on the complete 100-case suite****.*** *Macro F1 of 0.895 across represented categories is consistent with the overall accuracy, given the imbalanced class distribution in the evaluation suite.*

**Supplementary Table S7. Implementation Logic and Parameter Specification for Novel TeleMed CDSS v2.5 Pipeline Components**

Language-agnostic algorithmic logic for the four novel components of the TeleMed CDSS v2.5 prediction pipeline. These descriptions are sufficient to guide re-implementation of the core mechanisms. Full source code is not publicly available as it constitutes part of the ongoing ICMR clinical project (Data and Code Availability statement, main manuscript).

**Component 1: Decimal Positioning Two-Stage Decomposition**

| Step | Pseudocode | Purpose |
| --- | --- | --- |
| 1 | Encode class labels: tens_label ← y // 10; units_label ← y mod 10 | Decompose multi-class problem into two sub-problems within TabPFN's 10-class ceiling |
| 2 | Train TabPFN_tens on (X, tens_label); Train TabPFN_units_per_bucket on (X_bucket, units_label) for each tens bucket | Separate classifiers for coarse and fine prediction stages |
| 3 | At inference: p_tens ← TabPFN_tens.predict_proba(x); top_bucket ← argmax(p_tens) | Coarse-stage prediction selects the most probable tens bucket |
| 4 | p_units ← TabPFN_units[top_bucket].predict_proba(x); final_class ← top_bucket × 10 + argmax(p_units) | Fine-stage prediction within selected bucket; combined to produce final class index |
| 5 | confidence ← max(p_tens) × max(p_units); if confidence < threshold → trigger low_confidence_alert() | Combined confidence used for abstention/alert logic |
| 6 | valid_mask ← class_indices ≤ 20; p_final[~valid_mask] ← 0; final_class ← argmax(p_final) | Invalid class indices above 20 were masked and assigned zero probability before final class selection |

**Component 2: MiniLM Cosine Similarity Semantic Symptom Normalisation**

| Step | Pseudocode | Purpose |
| --- | --- | --- |
| 1 | Load encoder: model ← SentenceTransformer('all-MiniLM-L6-v2') | 384-dimensional sentence embedding model |
| 2 | Precompute: vocab_embeddings ← model.encode(training_symptom_vocabulary) | Encode standardised training vocabulary once at deployment |
| 3 | At inference: input_embedding ← model.encode(raw_symptom_text) | Encode incoming free-text symptom input |
| 4 | similarities ← cosine_similarity(input_embedding, vocab_embeddings) | Compute similarity to all vocabulary entries |
| 5 | best_match ← vocab[argmax(similarities)]; score ← max(similarities) | Retrieve highest-similarity vocabulary term |
| 6 | if score ≥ 0.45: normalised_symptom ← best_match; else: normalised_symptom ← raw_symptom_text | Threshold ≥ 0.45 applied: accepted if similarity sufficient, else raw text passed through unchanged |

**Component 3: Contextual Buffer Refitting Procedure**

| Step | Pseudocode | Purpose |
| --- | --- | --- |
| 1 | Maintain: context_buffer ← deque(maxlen = MAX_CONTEXT_SIZE); MAX_CONTEXT_SIZE = 8,000 | FIFO sliding window; oldest record evicted when ceiling reached |
| 2 | On doctor validation: context_buffer.append(verified_record); log(timestamp, doctor_id, correction) | Only doctor-approved records enter the buffer; all corrections logged |
| 3 | Rebuild: symptom_index ← encode_all(context_buffer.symptoms) | Re-encode symptom index for KNN retrieval from updated buffer |
| 4 | Refit: TabPFN_tens.fit(context_buffer.X, context_buffer.tens_labels); TabPFN_units.fit(...) for each bucket | Classifier heads refit on updated buffer; base model weights remain FROZEN |
| 5 | No backpropagation; no gradient update; inference immediately available post-refit | Zero downtime; no risk of catastrophic forgetting in base model parameters |

**Component 4: Multi-Layer Clinical Validation Logic**

| Step | Pseudocode | Purpose |
| --- | --- | --- |
| 1 | domain_boost ← min(1.0 + 0.5 × count_matching_domain_tokens(symptom, category), 3.0) | Capped linear boost applied to category probabilities; ceiling prevents over-weighting keyword-heavy inputs |
| 2 | boosted_probs ← softmax(raw_probs × domain_boost_vector) | Re-normalise after boosting |
| 3 | knn_results ← retrieve_top_k(symptom_index, query_embedding, k=5, weight = 0.7×symptom_sim + 0.3×(1−numeric_dist)) | KNN retrieval for high-cardinality outputs (Diagnosis, Medicines, Advice); combined semantic and physiological scoring |
| 4 | diagnosis ← knn_results[0].diagnosis; medicines ← knn_results[0].medicines; advice ← knn_results[0].advice | Top-1 retrieved record provides high-cardinality output fields |
| 5 | if 'antibiotic' in medicines and not antibiotic_flag_cleared: trigger antibiotic_safety_alert() | Antibiotic safety flag: first-layer pharmacovigilance safeguard |
| 6 | if max(boosted_probs) < confidence_threshold: trigger low_confidence_alert(); recommend_clinical_review() | Abstention-equivalent: uncertain predictions flagged for enhanced physician review rather than acted upon autonomously |
| 7 | All outputs presented in editable interface; doctor must explicitly save/approve before e-prescription generation | Mandatory doctor-in-the-loop gate; no autonomous prescription issuance |

*Pseudocode is language-agnostic and simplified for clarity. Actual implementation uses Python 3.10+, PyTorch-based TabPFN 2.5, sentence-transformers library, and scikit-learn KNN. All novel components operate within the FastAPI REST endpoint (POST /predict) accessible within the local network of the telemedicine facility.*

**Supplementary Table S8. Cross-validated per-class performance for parent-category classification**

| **Diagnostic Category** | **Development Records (n)** | **Evaluability Status** | **Precision** | **Recall** | **F1 Score** | **Note** |
| --- | --- | --- | --- | --- | --- | --- |
| Respiratory | 1,065 | Primary evaluable | 0.94 | 0.93 | 0.93 | High-frequency class |
| Gastrointestinal | 629 | Primary evaluable | 0.92 | 0.91 | 0.91 | High-frequency class |
| Musculoskeletal | 556 | Primary evaluable | 0.90 | 0.89 | 0.89 | High-frequency class |
| General Symptoms | 495 | Primary evaluable | 0.88 | 0.87 | 0.87 | High-frequency class |
| Neurological / Psychiatric | 470 | Primary evaluable | 0.76 | 0.75 | 0.75 | Lower performance; confusion with MSK/GI |
| General Symptoms -Seasonal | 304 | Primary evaluable | 0.89 | 0.88 | 0.88 | Moderate-frequency class |
| Dermatological | 258 | Primary evaluable | 0.91 | 0.90 | 0.90 | Moderate-frequency class |
| Dental / Oral | 131 | Primary evaluable | 0.89 | 0.88 | 0.88 | Lower-frequency class |
| Infectious Disease | 107 | Primary evaluable | 0.84 | 0.83 | 0.83 | Lower-frequency class |
| Eye and Vision | 94 | Primary evaluable | 0.87 | 0.85 | 0.86 | Lower-frequency class |
| Cardiovascular | 80 | Primary evaluable | 0.83 | 0.81 | 0.82 | Lower-frequency class |
| Injury / Trauma | 63 | Primary evaluable | 0.85 | 0.84 | 0.84 | Lower-frequency class |
| ENT | 46 | Primary evaluable | 0.82 | 0.80 | 0.81 | Lower-frequency class |
| Reproductive / Genitourinary | 41 | Primary evaluable | 0.80 | 0.78 | 0.79 | Lower-frequency class |
| Cardiovascular & Gastrointestinal | 39 | Primary evaluable | 0.76 | 0.74 | 0.75 | Compound category |
| Gastrointestinal & Musculoskeletal | 8 | Primary evaluable | 0.65 | 0.62 | 0.63 | Low-frequency compound |
| Cardiovascular & Endocrinal | 5 | Threshold / descriptive | 0.58 | 0.55 | 0.56 | Interpreted descriptively only |
| Respiratory & Gastrointestinal | 5 | Threshold / descriptive | 0.55 | 0.52 | 0.53 | Interpreted descriptively only |
| Endocrinal | 4 | Non-evaluable | - | - | - | Below minimum threshold (<5) |
| Respiratory & Dental | 1 | Non-evaluable | - | - | - | Below minimum threshold (<5) |

**Supplementary Table S9. Calibration and Low-Confidence Threshold Behaviour**

| **Metric / Stratum** | **Value** | **Interpretation** |
| --- | --- | --- |
| Overall Accuracy | 87.2% | Baseline cross-validated performance |
| Brier Score | 0.142 | Overall probabilistic accuracy |
| Expected Calibration Error (ECE) | 0.054 | Low divergence between confidence and accuracy |
| Low-Confidence Threshold | < 0.45 | Maximum predicted probability trigger for alert |
| Proportion Flagged | 14.5% | Encounters routed for enhanced physician review |
| Error Rate (Flagged Cases) | 38.5% | High uncertainty subset (61.5% accuracy) |
| Error Rate (Non-Flagged Cases) | 8.4% | High confidence subset (91.6% accuracy) |

*Note: The threshold of 0.45 was selected to balance clinical workload with patient safety. The stratified error rates demonstrate that the low-confidence alert successfully concentrates the majority of model uncertainty into the 14.5% of cases flagged for mandatory doctor oversight.*
